# tidytof: a user-friendly framework for scalable and reproducible high-dimensional cytometry data analysis

**DOI:** 10.1093/bioadv/vbad071

**Published:** 2023-06-09

**Authors:** Timothy J Keyes, Abhishek Koladiya, Yu-Chen Lo, Garry P Nolan, Kara L Davis

**Affiliations:** Medical Scientist Training Program, Stanford University School of Medicine, Stanford, CA 94305, USA; Department of Biomedical Data Science, Stanford University School of Medicine, Stanford, CA 94305, USA; Department of Pediatrics, Stanford University School of Medicine, Stanford, CA 94305, USA; Department of Pediatrics, Stanford University School of Medicine, Stanford, CA 94305, USA; Department of Pediatrics, Stanford University School of Medicine, Stanford, CA 94305, USA; Department of Pathology, Stanford University School of Medicine, Stanford, CA 94305, USA; Department of Pediatrics, Stanford University School of Medicine, Stanford, CA 94305, USA; Center for Cancer Cell Therapy, Stanford University School of Medicine, Stanford, CA 94305, USA

## Abstract

**Summary:**

While many algorithms for analyzing high-dimensional cytometry data have now been developed, the software implementations of these algorithms remain highly customized—this means that exploring a dataset requires users to learn unique, often poorly interoperable package syntaxes for each step of data processing. To solve this problem, we developed {tidytof}, an open-source R package for analyzing high-dimensional cytometry data using the increasingly popular ‘tidy data’ interface.

**Availability and implementation:**

{tidytof} is available at https://github.com/keyes-timothy/tidytof and is released under the MIT license. It is supported on Linux, MS Windows and MacOS. Additional documentation is available at the package website (https://keyes-timothy.github.io/tidytof/).

**Supplementary information:**

Supplementary data are available at *Bioinformatics Advances* online.

## 1 Introduction

**Figure 1.**
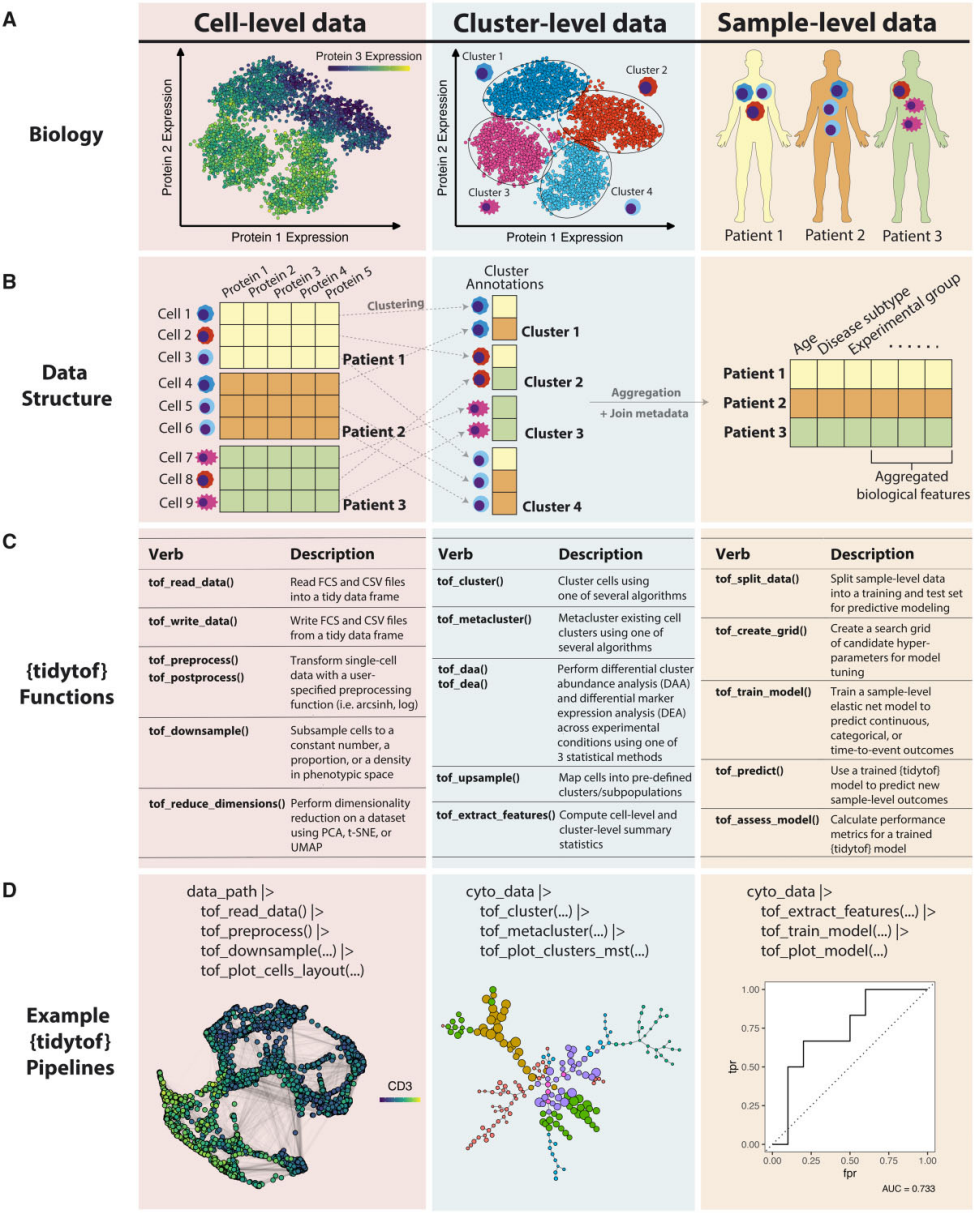
{tidytof} implements a ‘grammar’ of single-cell data analysis at the single-cell, cluster and whole-sample levels. (**A**) Mass cytometry data contains biological insights at multiple levels of observation. The cell level (left) contains information about individual cells’ marker expression profiles. The cluster level (middle) contains information about the cell subpopulations that predominate among single-cell phenotypes. The whole-sample level (right) contains information about patient outcomes or experimental conditions that can be associated with single-cell and cluster-level information. (**B**) ‘Tidy’ data structures can be used to represent high-dimensional cytometry data at the single-cell, cluster and sample levels. At the cell level (left), a tidy data frame represents each cell as a row and each protein measurement as a column (each square in the image represents a single numeric measurement). Cells from multiple samples can be grouped by their sample-of-origin (represented using the color of each row) and bound row-wise into a single tidy data frame (see Supplementary Table S1 for an expanded representation). At the cluster level (middle), cluster membership can be represented by assigning a cluster identifier to each cell, allowing cells to be regrouped according to cluster membership. Finally, information from the single-cell and cluster levels can then be aggregated by computing summary statistics (such as each cluster’s abundance and mean marker expression) and joined with sample-level metadata to form a tidy data frame in which each row represents a sample and each column represents an aggregated biological feature or piece of metadata (right). (**C**) {tidytof} functions associated with the cell, cluster and whole-sample levels of data analysis. For expanded versions of these tables, see Supplementary Tables S2–S4. (**D**) Example code snippets that use {tidytof} functions to process and visualize high-dimensional cytometry data at the cell (left), cluster (middle) and whole-sample (right) levels. {tidytof} functions are chained together into mix-and-match data processing pipelines using R’s native pipe operator (‘|>’). In the snippets, ‘data_path’ represents a file path to input .fcs or .csv files, ‘cyto_data’ represents a high-dimensional cytometry dataset in tidy format, and **…** represents function-specific arguments abbreviated for clarity. See Supplementary vignettes for additional details

Over the past decade, high-dimensional cytometry has become a prominent technology for high-throughput single-cell analysis of both human and animal tissues (Spitzer and Nolan, 2016). Platforms including mass cytometry (or Cytometry by Time-Of-Flight), full-spectrum flow cytometry and sequence-based cytometry have now enabled the collection of large datasets of multiplexed proteomic measurements from millions of cells per experiment (Bendall *et al.*, 2011; Jaimes *et al.*, 2022; Keyes *et al.*, 2020; Salomon *et al.*, 2020). To derive insights from these complex datasets, recent years have also seen the development of dozens of algorithms for analyzing high-dimensional cytometry data at the single-cell, cell subpopulation and whole-sample levels (Fig. 1A) [for comprehensive reviews, see Keyes *et al.* (2020), Olsen *et al.* (2019) and Hu *et al.* (2022)]. However, navigating the selection, use and interoperability requirements for each of these methods remains a significant challenge for many biologists.

Within the same time-period, the concept of ‘tidy data’—i.e. data represented in flexible, two-dimensional tables (called *data frames*) in which each row represents an observation and each column represents an experimental variable—has constituted a paradigm shift within the field of data science (Fig. 1 and Supplementary Table S1) (Wickham, 2014). The central concept of data ‘tidiness’ is that representing data in a consistent, tidy format makes data processing simpler (and often faster) by standardizing the tools needed to build an analytical pipeline. As such, adopting tidy data practices generally encourages the use of intuitive, human-centered design principles in statistical software engineering, allowing researchers to apply similar analytical frameworks across tools and research domains by expressing common data processing operations using a self-consistent vocabulary. In the context of scientific computing, using tidy data is particularly powerful because it results in code that is easy-to-read (and thereby easy-to-maintain), making it error-resistant, reproducible and relatively easy to write and debug. Because of these useful qualities, tidy workflows are emerging for bioinformatics applications in genomic (Lee *et al.*, 2019) and transcriptomic analysis (Mangiola *et al.*, 2021a, b). Here, we build on this previous work by presenting {tidytof}, an R package implementing a comprehensive, performant and extensible framework for analyzing high-dimensional cytometry data with a tidy interface. Comprehensive documentation and tutorials for how to use {tidytof} are available in Supplementary Notes S1 and S2 and in the {tidytof} package vignettes.

## 2 Software design

{tidytof} consolidates popular methods of high-dimensional cytometry data analysis, such as file reading, preprocessing, batch correction, quality control, clustering, dimensionality reduction, differential expression analysis, feature extraction and visualization into a composable, easy-to-use application programming interface (API) suitable for both experienced and beginner programmers (see Supplementary Tables S2–S4). It does so by organizing common data analysis tasks—including state-of-the-art statistical analyses and machine-learning algorithms—into verbs, or modular function families that represent specific mathematical operations like dimensionality reduction and clustering (Fig. 1C). Each {tidytof} verb accepts user commands with the same structure: a tidy data frame is accepted as input, and a tidy data frame is returned as output. Specifically, {tidytof} stores high-dimensional cytometry data in a specialized data structure called a *tof_tbl* (Supplementary Table S1). *tof_tbl’*s are an extension of R’s *data.frame* class that are customized to store cytometry data in a tidy format: cells are represented as rows, protein (or transcript) measurements are represented as columns and metadata about the cytometry panel used during data acquisition is stored as a class attribute. In addition, *tof_tbl’*s can also incorporate columns (using tidytof verbs’ *augment* flag) that store additional information about each cell—e.g. a cluster assignment, a low-dimensional embedding, or metadata information about the sample from which the cell was collected. Unlike other data structures commonly used to store single-cell data (such as *SingleCellExperiment* or *flowSet* objects), *tof_tbl’*s directly inherit behavior from *data.frame’*s, enabling them to dispatch any of the many existing methods for the *data.frame* class (Amezquita *et al.*, 2020; Ellis *et al.*, 2021). Furthermore, a key feature of {tidytof}’s API is its ability to assemble multiple functions into modular, multistep pipelines that combine several analytical steps but remain easily human-readable by iteratively transforming a *tof_tbl* one step at a time (Fig. 1D and Supplementary Notes S1 and S2). Thus, tidytof stores cell-level data in the rows of a *tof_tbl*, allowing cluster- and sample-level data to be quickly aggregated using {tidytof} verbs’ grouped operations.

**Figure 2.**
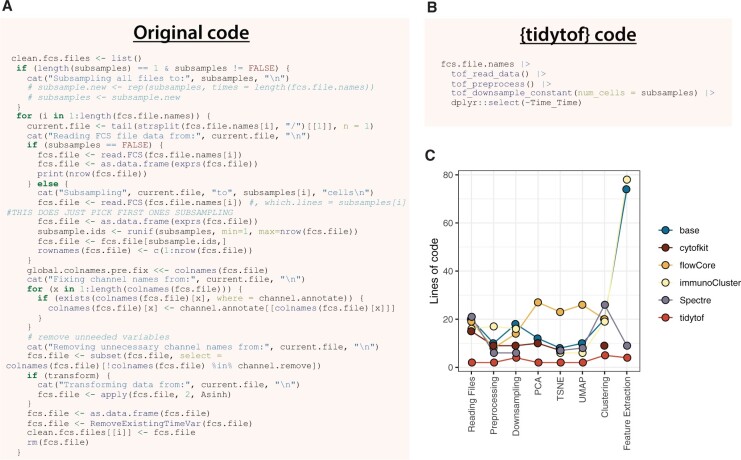
{tidytof} simplifies the code needed to perform high-dimensional cytometry data analysis. (**A**) Raw code from the body of LoadCleanFCS(), a function used for reading, preprocessing and downsampling FCS files in Ko *et al.* (2020). This code is replicated without editing, and the original source is available here: https://github.com/zunderlab/FLOWMAP/blob/master/R/load-from-FCS.R. Note that in the original function, the variable *fcs.file.names* is a character vector containing the file path to each FCS file to be read into memory, and the variable *subsamples* is an integer indicating how many cells to samples from each FCS file. (**B**) {tidytof} code that provides the same functionality as that provided in (**A**). *fcs.file.names* and *subsamples* are variables defined as in (A). (**C**) Raw lines of code needed to perform equivalent analyses using {tidytof} and other open-source single-cell data analysis packages, including base R, {cytofkit}, {flowCore}, {immunoCluster} and {Spectre}. Functions used to perform the code comparisons are provided at the following GitHub link: https://github.com/keyes-timothy/tidytof-manuscript/blob/72c2d347241660156b1751cfdad1f0849df3dccb/benchmarking/benchmarking_functions.R. For comprehensive coding burden comparisons with other packages, see Supplementary Figures S3 and S4

This design strategy has three major benefits. First, it allows {tidytof} to simplify how users access algorithms that perform the same fundamental task in different ways. For example, the *tof_cluster* verb provides a single interface to multiple clustering algorithms (see Supplementary Table S3) that, despite their distinct underlying implementations, can all be applied easily using {tidytof}’s consistently tidy input and output data formats. This means that users can focus their efforts on interpreting and refining the results of a data analysis pipeline rather than on maintaining large code bases, which {tidytof} can simplify into relatively few lines of code (Fig. 2). Second, {tidytof} verbs’ modular design means that they can be combined to perform flexible analyses at each level of biological scope inherent to single-cell data: the single-cell level, the cell subpopulation level and the whole-sample level. {tidytof} provides verbs for computation and visualization within each of these levels as well as for transforming data between them. This results in a concise and intuitive domain-specific language for single-cell data analysis designed to answer questions at multiple levels of biological interest (Fowler, 2010). Third, as an extension of the high-performing tidyverse ecosystem of data analysis tools in R, {tidytof} provides superior computational performance relative to existing packages for single-cell data analysis.

## 3 Performance benchmarking

**Figure 3.**
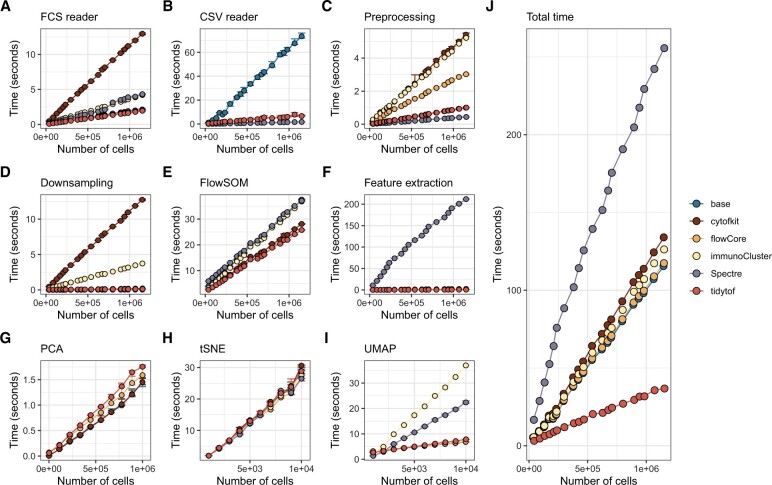
{tidytof}’s computational speed rivals or improves upon equivalent approaches using existing tools. Benchmark plots indicating the elapsed time for (**A**) reading FCS files, (**B**) reading CSV files, (**C**) preprocessing single-cell data, (**D**) downsampling single-cell data, (**E**) performing FlowSOM clustering, (**F**) sample-level feature extraction, (**G**) performing principal component analysis (PCA), (**H**) performing *t*-stochastic neighborhood embedding (tSNE), or (**I**) performing UMAP embedding using {tidytof}, base R, {flowCore}, {cytofkit}, {immunoCluster} and {Spectre}. Panel (**J**) shows the sum of runtimes from panels (A–F) for each tool [because panels (G–I) were run on different datasets, they could not be combined with the other panels] In all panels, points represent the median runtime for each workflow from 10 independent repetitions and error bars represent the interquartile range of runtimes

We benchmarked {tidytof}’s speed and memory performance against equivalent workflows in two low-level APIs (base R and {flowCore}) as well as three high-level APIs ({cytofkit}, {immunoCluster} and {Spectre}) with similar capabilities (Ashhurst *et al.*, 2021; Chen *et al.*, 2016; Ellis *et al.*, 2021; Opzoomer *et al.*, 2021; R Core Team, 2021). Workflows—which included reading input files, preprocessing, downsampling, clustering, dimensionality reduction and feature extraction—were evaluated on the basis of their computational speed and memory performance on datasets sampled from a publicly available cohort of mass cytometry samples (Good *et al.*, 2018). Across all workflows, {tidytof}’s computational performance rivaled or surpassed existing tools (Fig. 3A–I), and {tidytof}’s total time across all workflows was the smallest for all datasets used for benchmarking (Fig. 3J).

Notably, {tidytof}’s competitive performance is primarily attributable to efficient usage of the tidyverse package ecosystem, which interfaces easily with many other packages as an extension of the base R environment. This is exemplified by {tidytof}’s superior performance at file reading and downsampling compared to {cytofkit}, whose non-modular design results in redundant computations; by its superior performance at feature extraction compared to {Spectre}, which suffers a substantial performance penalty due to its use of for-loops; and its superior wrapping of uniform manifold approximation and projection (UMAP) compared to {immunoCluster} and {Spectre}, both of which are substantially slowed by their need to convert to and from *data.table* and *SingleCellExperiment* data objects, respectively. Despite this, {tidytof} is also as memory efficient as other tools for high-dimensional cytometry data analysis, suggesting that its speed does not derive from a tradeoff between data storage and computational performance (Supplementary Fig. S2). For comprehensive details and additional benchmarking, see the ‘{tidytof} performance benchmarking’ section of the Supplementary Material.

## 4 Conclusion

In summary, {tidytof} provides a tidy interface for analyzing high-dimensional cytometry data with an easy-to-use API and seamless integration with many existing tools created by the data science and bioinformatics communities (Wickham *et al.*, 2019). In doing so, {tidytof} decreases the coding burden of applying standard tools to high-dimensional cytometry datasets, thereby increasing the accessibility of advanced analytical methods to researchers with limited programming experience. While explicitly focused on cytometry data, {tidytof} also proposes a more general framework for creating a unified ‘grammar’ of single-cell data analysis—one that involves standard strategies for reading/writing data, preprocessing, clustering, feature engineering/aggregation and statistical modeling. This framework is widely extensible beyond cytometric technologies themselves. Our future engineering efforts will include both implementing new data processing features as additional verbs as well as extending existing verbs’ compatibility with other emerging single-cell technologies, such as high-dimensional imaging (Black *et al.*, 2021; Levenson *et al.*, 2015; Ståhl *et al.*, 2016).

## Supplementary Material

vbad071_Supplementary_Data

## Data Availability

Mass cytometry data used for performance benchmarking are available at https://github.com/kara-davis-lab/DDPR/releases.
