# Supplementary material for: tidytof: a user-friendly framework for scalable and reproducible high-dimensional cytometry data analysis
Source: Bioinform Adv. 2023 Jun 9;3(1):vbad071. doi: 10.1093/bioadv/vbad071 (PMC10281957; doi:10.1093/bioadv/vbad071)
Supplement: vbad071_Supplementary_Data [file vbad071_supplementary_data.pdf]

{tidytof}: A user-friendly framework for scalable and reproducible high-dimensional cytometry data analysis

Supplementary Information

Timothy Keyes, Abhishek Koladiya, Yu Chen Lo, Garry P. Nolan, and Kara L. Davis

2023-05-03

Contents

Supplementary Tables **2**
Supplementary Table 1 . . . 2
Supplementary Table 2 . . . 3
Supplementary Table 3 . . . 4
Supplementary Table 4 . . . 5

{tidytof} performance benchmarking **6**
Methods . . . 6
Benchmarking datasets . . . 6
Speed benchmarking . . . 7
Memory benchmarking . . . 7
Coding burden comparison . . . 7
Results . . . 8
Speed benchmarking (Supplementary Figure 1) . . . 8
Memory benchmarking (Supplementary Figure 2) . . . 10
Coding burden comparison (Supplementary Figures 3-4) . . . 11

Supplementary Notes **14**
Supplementary Note 1 - A beginner's introduction to {tidytof} . . . 14
Prerequisites . . . 14
Workflow basics . . . 15
{tidytof} verb syntax . . . 15
Pipelines . . . 17
Other tips . . . 18
Supplementary Note 2 - Example {tidytof} workflows . . . 20
Accessing the data for this vignette . . . 20
Analyzing single-cell data . . . 21
Analyzing cluster-level data . . . 24
Analyzing sample-level data . . . 26

References **28**

Supplementary Tables

Supplementary Table 1

	Proteomic data			{tidytof} calculations			Metadata	
**cell_id**	**protein_1**	**protein_2**	**protein_3**	**tsne_1**	**tsne_2**	**cluster**	**sample_type**	**patient**
Cell 1	0.05	1.55	-0.04	13.68	6.28	1	healthy	patient 1
Cell 2	1.33	2.04	1.38	14.67	-2.37	2	healthy	patient 1
Cell 3	-0.20	-0.16	-0.03	-2.11	8.51	4	healthy	patient 1
Cell 4	1.23	1.64	0.41	-3.64	16.77	1	healthy	patient 2
Cell 5	0.35	0.10	0.00	-5.28	14.29	4	healthy	patient 2
Cell 6	0.05	-0.20	-0.20	1.19	-3.63	4	healthy	patient 2
Cell 7	2.08	-0.07	0.61	-4.58	15.79	3	cancer	patient 3
Cell 8	2.77	2.16	2.05	12.67	-8.24	2	cancer	patient 3
Cell 9	1.40	0.36	-0.06	-1.40	-7.27	3	cancer	patient 3

Supplementary Table 1 - Example of a `{tidytof}` data frame. {tidytof} represents high-dimensional cytometry data in a "tidy format" using an extended data frame called a "tof_tbl". In this format, data are represented such that each cell is given its own row and each measurement or piece of metadata is given its own column.

Supplementary Table 2

Verb	Family members	Description
**tof_read_data()**	• tof_read_fcs()<br>• tof_read_csv()	Read FCS and CSV files on disk into a tidy data frame.
**tof_write_data()**	• tof_write_fcs()<br>• tof_write_csv()	Write FCS and CSV files to disk from a tidy data frame.
**tof_transform()**<br>**tof_preprocess()**<br>**tof_postprocess()**		Transform single-cell data with a user-specified function (i.e. variance-stabilizing transformation, scaling, denoising, etc.).
**tof_downsample()**	• tof_downsample_constant()<br>• tof_downsample_prop()<br>• tof_downsample_density()	Subsample cells to a constant number, to a proportion of the cells in the input, or to an estimated density in phenotypic space [1].
**tof_reduce_dimensions()**	• tof_reduce_pca()<br>• tof_reduce_tsne()<br>• tof_reduce_umap()	Perform dimensionality reduction on a dataset using principal components analysis (PCA), t-stochastic neighborhood embedding (tSNE) [2], or uniform manifold approximation and projection (UMAP) [3].
**tof_assess()**	• tof_assess_channels()<br>• tof_assess_flow_rate()	Evaluate the staining quality of cytometry channels and identify periods of abnormally low or high flow rates during data acquisition.

Supplementary Table 2 - Cell-level verbs. A comprehensive table of `{tidytof}` verbs that operate at the single-cell level.[1–3]

Supplementary Table 3

Verb	Family members	Description
**tof_cluster()**	• tof_cluster_ddpr()<br>• tof_cluster_flowsom()<br>• tof_cluster_kmeans()<br>• tof_cluster_phenograph()<br>• tof_cluster_xshift()	Assign cluster labels to each cell in a dataset using one of several clustering algorithms commonly applied to high-dimensional cytometry data [4-7].
**tof_metacluster()**	• tof_metacluster_consensus()<br>• tof_metacluster_flowsom()<br>• tof_metacluster_hierarchical()<br>• tof_metacluster_kmeans()<br>• tof_metacluster_phenograph()	Agglomerate clusters into a smaller, user-specified number of metaclutsters using several common methods. [5-6; 8]
**tof_analyze_abundance()**	• tof_analyze_abundance_diffcyt()<br>• tof_analyze_abundance_glmm()<br>• tof_analyze_abundance_ttest()	Perform differential abundance analysis (DAA) of clusters across experimental conditions using one of 3 statistical methods. [9-10]
**tof_analyze_expression()**	• tof_analyze_expression_diffcyt()<br>• tof_analyze_expression_lmm()<br>• tof_analyze_expression_ttest()	Perform differential expression analysis (DEA) of clusters across experimental conditions using one of 3 statistical methods. [9-10]
**tof_extract_features()**	• tof_extract_central_tendency()<br>• tof_extract_proportion()<br>• tof_extract_threshold()<br>• tof_extract_emd()<br>• tof_extract_jsd()	Aggregate cluster-level information across single cells to compute summary statistics including the proportion of cells in each cluster, marker expression central tendencies (i.e. mean, median), and the proportion of cells with marker expression over a given threshold. [11]
**tof_upsample()**	• tof_upsample_distance()<br>• tof_upsample_neighbor()	Map each cell in a dataset to its most similar cluster in a set of predefined clusters. Useful if clustering was performed on a downsampled dataset for computational efficiency, but cluster labels are needed for the full dataset. [1]
**tof_assess_clusters()**	• tof_assess_clusters_distance()<br>• tof_assess_clusters_entropy()	Evaluate a clustering result by flagging cells that are unusually far from the centroid of the cluster to which they were assigned or by flagging cells who assignment to any particular cluster is ambiguous.

Supplementary Table 3 - Cluster-level verbs. A comprehensive table of `{tidytof}` verbs that operate at the cluster level.[1, 4–11]

Supplementary Table 4

Verb	Description
**tof_split_data()**	Split sample-level data into a training and test set for predictive modeling (including k-fold cross-validation and bootstrapped resampling). [12]
**tof_create_grid()**	Create a search grid of candidate hyperparameters to test during model tuning.
**tof_train_model()**	Train a sample-level elastic net model to predict continuous (linear regression), categorical (logistic and multinomial regression), or time-to-event (Cox proportional-hazards regression) outcomes. [13-14]
**tof_predict()**	Apply a trained {tidytof} model to a new dataset to predict sample-level outcomes. [13-14]
**tof_assess()**	Interrogate a trained {tidytof} model's performance by calculating evaluation metrics. [13-14]

Supplementary Table 4 - Sample-level verbs. A comprehensive table of `{tidytof}` verbs that operate at the whole-sample level. [12–14]

{tidytof} performance benchmarking

Methods

To benchmark {tidytof}'s performance against existing tools for high-dimensional cytometry data analysis, we compared {tidytof} functions to their equivalent workflows (where they existed) using two low-level APIs (base R [15] and {flowCore} [16]) and three high-level APIs ({cytofkit} [17], {immunoCluster} [18], and {Spectre} [19]) with similar capabilities. Workflows were chosen to represent common high-dimensional cytometry data processing tasks including reading input files, data preprocessing, downsampling, clustering, dimensionality reduction, and sample-level feature extraction (i.e. calculating summary statistics).

Workflows were compared both on the basis of their computational speed and on the basis of their coding burden (i.e. the amount of code needed for each workflow). Specifically, comparisons included one metric for computational speed (total elapsed time for each workflow) and two metrics for coding burden associated with human error when conducting computational analyses (the number of lines of code and the number of intermediate variable assignments needed for the workflow). In addition, {tidytof}'s memory requirements were benchmarked against those of the {flowCore} package's `flowSet` objects - a commonly-used data structure for the analysis of high-dimensional cytometry data - as well as against {cytofkit} `data.frames`, {immunoCluster} `SingleCellExperiments` [20], and {Spectre} `data.tables` [21].

All benchmarking was performed on a Linux machine running Ubuntu 20.04 with an AMD Threadripper Pro 3995WX processor (64 cores; 2.70 GHz; 256 MB cache) and 256 GB of RAM. All functions used to perform speed, coding burden, and memory benchmarking are available here.

Benchmarking datasets

The data used for benchmarking were drawn from Good et al.'s publicly available CyTOF dataset published in 2018 [4]. Specifically, 20 files acquired from 2 healthy patients were chosen from the full dataset, totaling 1151293 cells. The following files were used:

File name
Healthy1_Basal.fcs
Healthy1_BCR-Crosslink.fcs
Healthy1_IL-7.fcs
Healthy1_TSLP.fcs
Healthy2_Basal_Part1.fcs
Healthy2_Basal_Part2.fcs
Healthy2_Basal_Part3.fcs
Healthy2_Basal_Part4.fcs
Healthy2_BCR-Crosslink_Part1.fcs
Healthy2_BCR-Crosslink_Part2.fcs
Healthy2_BCR-Crosslink_Part3.fcs
Healthy2_BCR-Crosslink_Part4.fcs
Healthy2_IL-7_Part1.fcs
Healthy2_IL-7_Part2.fcs
Healthy2_IL-7_Part3.fcs
Healthy2_IL-7_Part4.fcs

File name
Healthy2_TSLP_Part1.fcs
Healthy2_TSLP_Part2.fcs
Healthy2_TSLP_Part3.fcs
Healthy2_TSLP_Part4.fcs

Speed benchmarking

For speed benchmarking, each workflow was run on 20 nested datasets derived from the files listed above such that each dataset i was comprised of the first i files in the list. Thus, the smallest dataset included only 1 file (and 38382 cells) while the largest dataset included all 20 files (and 1151293 cells). However, due to the computationally intensive nature of t-stochastic neighborhood embedding (tSNE) and uniform manifold approximation and projection (UMAP), speed benchmarking was performed using 10 subsampled versions of the full dataset (all 20 files) with a smaller number of cells drawn at random: 1K, 2K, 3K, 4K, 5K, 6K, 7K, 8K, 9K, and 10K.

Using these datasets, the `microbenchmark` package [22] was used to time each workflow independently on each dataset 10 times. The median and interquartile range of each workflow's runtime could then be computed for {tidytof} and each alternative tool. For dimensionality reduction and clustering analyses, only cluster of differentiation ("CD") markers were used for analysis. For downsampling, 200 cells were subsampled from each input FCS or CSV file. Otherwise, {tidytof}'s default behavior was used across all benchmarking functions.

Memory benchmarking

For memory benchmarking, each nested dataset described above was read into each of the following:

- A `tof_tbl` object using {tidytof}'s `tof_read_data()` function
- A `flowSet` object using {flowCore}'s `read.flowSet()` function
- A `data.frame` using {cytofkit}'s `cytof_exprsMerge()` function
- A `SingleCellExperiment` object using {immunoCluster}'s `processFCS()` function
- A `data.table` using {Spectre}'s `read.files()` function.

The system memory used to store each object was then calculated using the `obj_size()` function from the {lobstr} package [23].

Coding burden comparison

For coding burden comparisons, the source code of the functions containing each workflow was subjected to text analysis. To control for differences in code style and practices between workflows, the {styler} package [24] was used to standardize all benchmarking code to adhere to the tidyverse R code style guide. The functions used to count the number of lines of code and the number of variable assignments in each workflow are provided here:

```
# count the lines of code for a given workflow
get_lines <- function(function_object) {
  if (is.na(function_object)) {
    return(NA)
  } else {
    lines <- capture.output(print(function_object))
    # substract 2 lines to omit the function definition and closing
    # bracket for each workflow
    return(length(lines) - 2L)
  }
```

```
}

# count the number of assigned variables for a given workflow
get_assignments <- function(function_object) {
  if (is.na(function_object)) {
    return(NA)
  } else {
    lines <- capture.output(print(function_object))
    assignments <- max(sum(str_count(lines, pattern = "<-")), 1L)
    return(assignments)
  }
}
```

For both functions above, the input is a function (`function_object`) used to define a {tidytof}, base R, {flowCore}, {cytofkit}, {immunoCluster}, or {Spectre} workflow, and the output is an integer representing the number of lines of code or the number of variable assignments, respectively, contained in that workflow. The functions used to define each workflow are available on GitHub here.

Results

Speed benchmarking (Supplementary Figure 1)

Across a wide variety of workflows including FCS and CSV file reading, single-cell data transformation (preprocessing), downsampling, FlowSOM clustering, sample-level feature extraction, tSNE embedding, and UMAP embedding, {tidytof}'s time efficiency rivals or improves upon alternative approaches (**Supplementary Figure 1**). In the case of PCA, {tidytof} incurs a slight performance decrease relative to alternative tools because it uses the `{embed}` package [25] to store a dataset's PCA embedding as a `recipe` object (from the {recipes} package) [26] - which, while costing a small amount of computational overhead, allows users to more easily apply the same embedding to new data points. While {Spectre}'s use of `data.table` makes it slightly faster than {tidytof} for simple arithmetic tasks like data preprocessing (**Supplementary Figure 1C**) and downsampling (**Supplementary Figure 1D**), {tidytof} requires substantially less cumulative time than competing methods when performing multiple workflows (**Supplementary Figure 1J**).

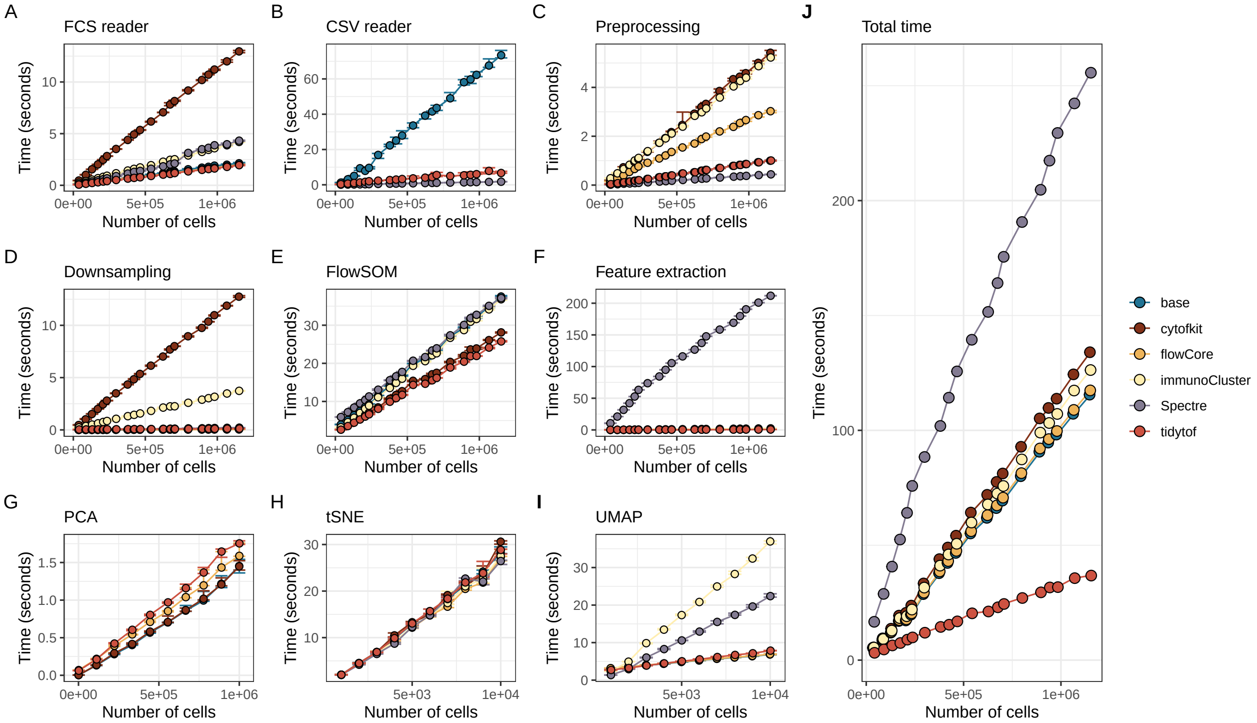

Supplementary Figure 1 - {tidytof}'s computational speed rivals or improves upon equivalent approaches using existing tools. Benchmark plots indicating the elapsed time for **(A)** reading FCS files, **(B)** reading CSV files, **(C)** preprocessing single-cell data, **(D)** downsampling single-cell data, **(E)** performing FlowSOM clustering, **(F)** sample-level feature extraction, **(G)** performing principal component analysis (PCA), **(H)** performing t-stochastic neighborhood embedding (tSNE), or **(I)** performing uniform manifold approximation and projection (UMAP) embedding using {tidytof}, base R, {flowCore}, {cytofkit}, {immunoCluster}, and {Spectre}. Panel **(J)** shows the sum of runtimes from panels A-F for each tool (because panels G-I were run on different datasets, they could not be combined with the other panels). In all panels, points represent the median runtime for each workflow from 10 independent repetitions and error bars represent the interquartile range of runtimes.

Memory benchmarking (Supplementary Figure 2)

Despite the increased simplicity of {tidytof}'s tidy data structures (`tof_tbl` objects) compared to {flowCore}'s native data structure (the `flowSet`), both data structures require similar amounts of system memory (**Supplementary Figure 2**). Furthermore, {tidytof} requires slightly less memory than {Spectre} `data.table`s and substantially less memory than {immunoCluster} `SingleCellExperiment`s and {cytofkit} `data.frame`s across all dataset sizes.

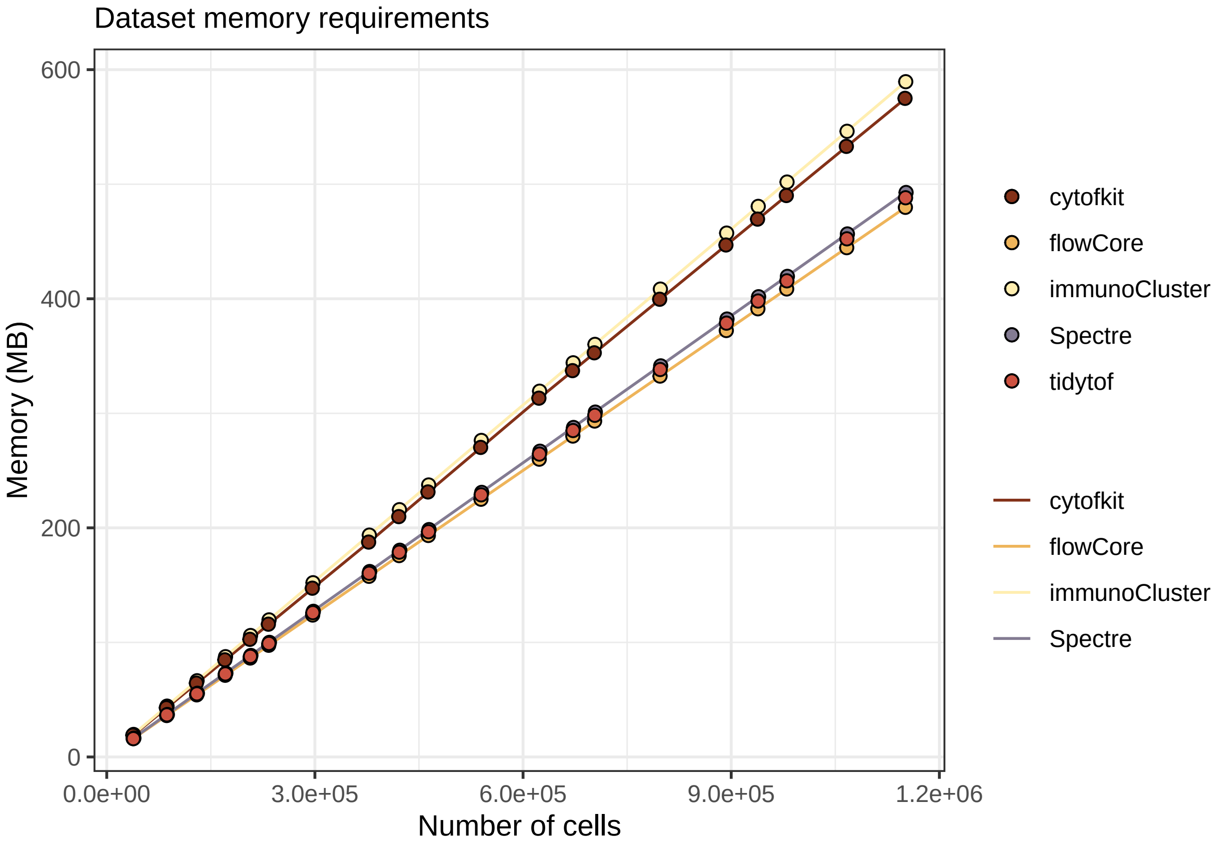

Supplementary Figure 2 - {tidytof}'s tidy data structures are memory-efficient compared to existing software tools The system memory (in Megabytes; MB) required to store {tidytof}, {flowCore}, {cytofkit}, {immunoCluster}, and {Spectre} data structures of varying sizes. Overall, {tidytof} is competitive with {flowCore} and {Spectre} while offering substantial improvements in memory efficiency relative to {cytofkit} and {immunoCluster}.

Coding burden comparison (Supplementary Figures 3-4)

In addition to its competitive computational speed relative to equivalent workflows using other cytometry data analysis software, {tidytof} reduces the coding burden of performing high-dimensional cytometry data analyses significantly compared to alternative frameworks. Across workflows, {tidytof} reduces the lines of code needed to perform an analysis between 33% and 95% compared to alternative software tools (**Supplementary Figure 3**). Similarly, {tidytof} reduces the number of variable assignments needed to perform an analysis between 67% to 98% compared alternative software tools (**Supplementary Figure 4**). Because code bases with both more lines of code and more intermediate variables are more prone to human (e.g copy-and-paste) error and reduced readability, these results support {tidytof}'s marked ability to simplify high-dimensional cytometry data analysis code and increase reproducibility among data analysis pipelines.

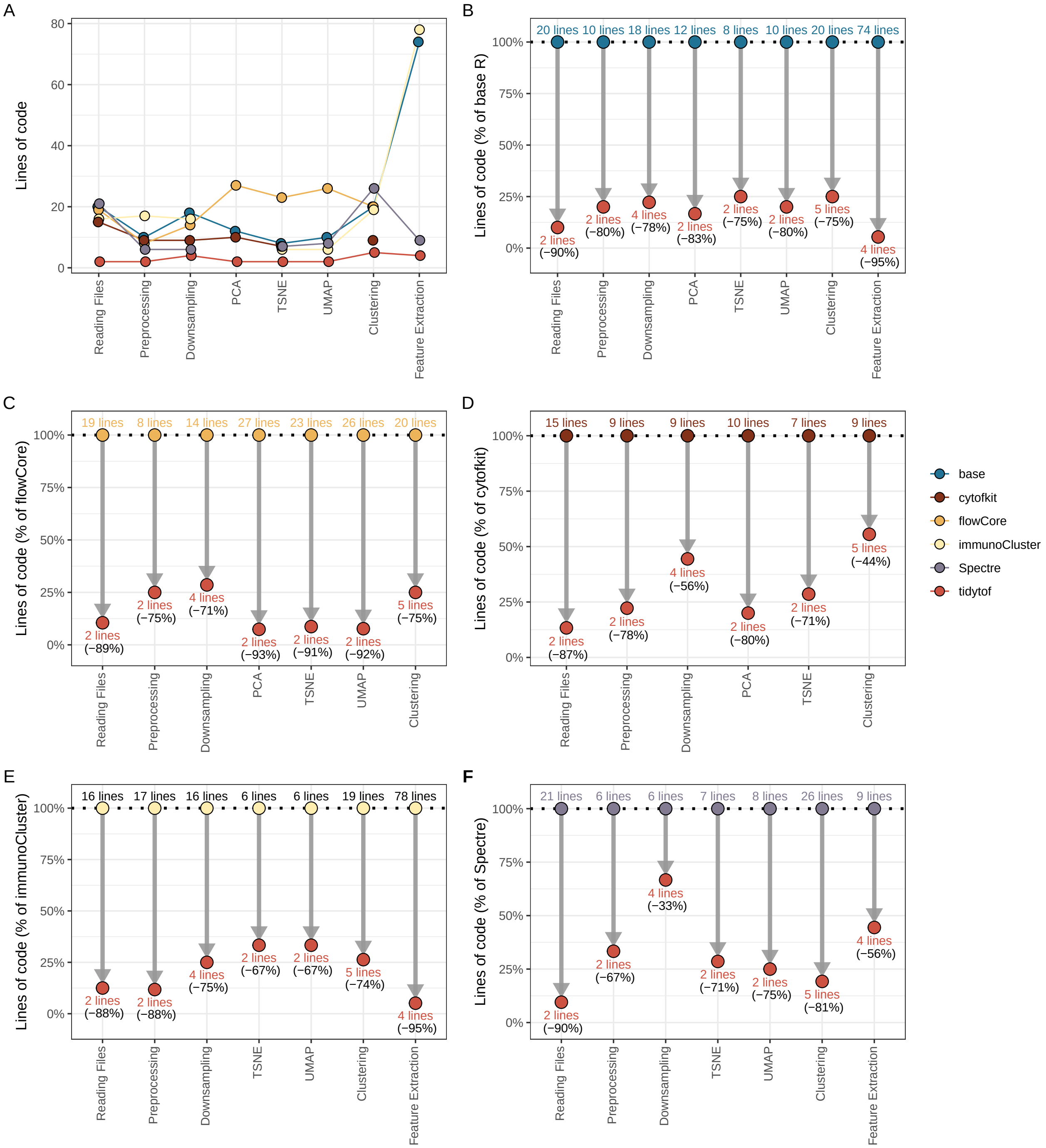

Supplementary Figure 3 - {tidytof} reduces the number of lines of code needed for high-dimensional cytometry data analysis pipelines. (A) Raw lines of code needed to perform equivalent analyses using {tidytof} and other open-source single-cell data analysis platforms. **(B-F)** The relative (%) lines of code needed to perform equivalent analyses between {tidytof} and base R (B); between {tidytof} and {flowCore} (C); between {tidytof} and {cytofkit} (D); between {tidytof} and {immunoCluster} (E); and between {tidytof} and {Spectre} (F).

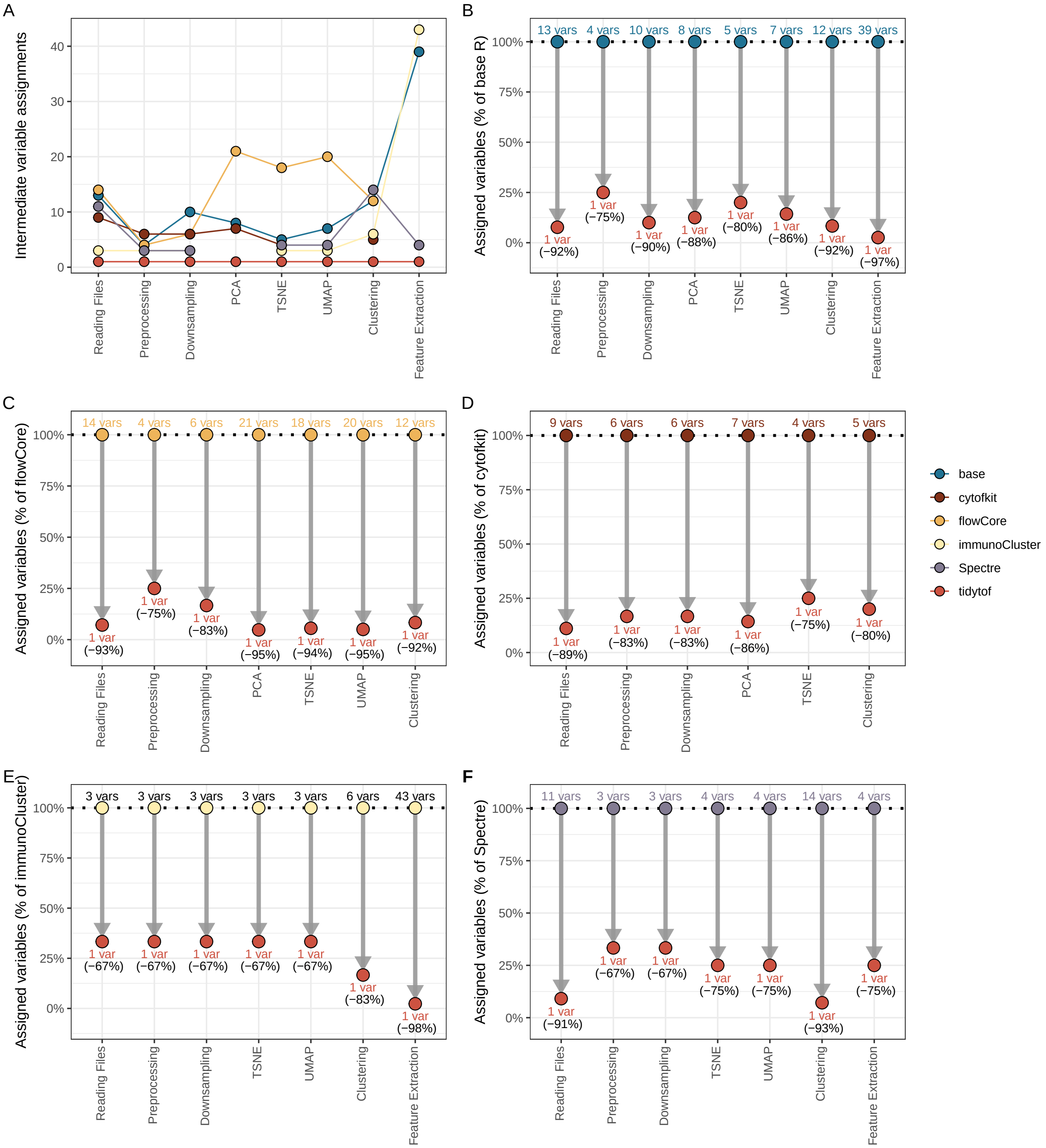

Supplementary Figure 4 - {tidytof} reduces the number of intermediate variable assignments needed for high-dimensional cytometry data analysis pipelines. (A) Raw number of intermediate variable assignments needed to perform equivalent analyses using {tidytof} and other open-source single-cell data analysis platforms. **(B-F)** The relative (%) number of intermediate variable assignments needed to perform equivalent analyses between {tidytof} and base R (B); between {tidytof} and {flowCore} (C); between {tidytof} and {cytofkit} (D); between {tidytof} and {immunoCluster} (E); and between {tidytof} and {Spectre} (F).

Supplementary Notes

Supplementary Note 1 - A beginner's introduction to {tidytof}

Analyzing single-cell data can be surprisingly complicated. One one hand, this is partially because single-cell data analysis is an incredibly active area of research, with new methods being published at a rapid rate. Accordingly, when new tools are shared with the scientific community, they often require researchers to learn unique, method-specific application programming interfaces (APIs) with distinct requirements for input data formatting, function syntax, and output data structure. Furthermore, analyzing single-cell data can be challenging because it often involves simultaneously asking questions at multiple levels of biological scope - the single-cell level, the cell subpopulation (i.e. cluster) level, and the whole-sample or whole-patient level - each of which has distinct data processing needs.

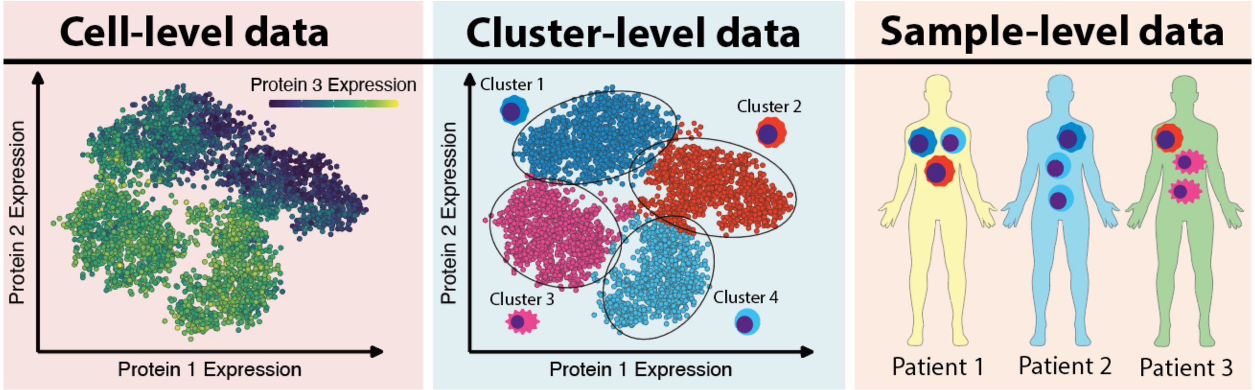

Figure caption: *Researchers are often interested in biological questions that manifest at multiple levels of single-cell data processing. At the single-cell level, you may be interested in individual cells' marker expression profiles. At the cluster level, you may be interested in how cells organize into subpopulations with shared phenotypic characteristics of varying size and similarity. And at the whole-sample level, you may be interested in how individual cells' and clusters' characteristics manifest in disease, treatment response, or a variety of other clinical or experimental variables.*

To address both of these challenges for high-dimensional cytometry [27], `{tidytof}` implements a concise, integrated "grammar" of single-cell data analysis capable of answering a variety of biological questions. Available as an open-source R package, `{tidytof}` provides an easy-to-use pipeline for analyzing high-dimensional cytometry data by automating many common data-processing tasks under a common "tidy data" interface. This vignette introduces you to the tidytof's high-level API and shows quick examples of how they can be applied to high-dimensional cytometry datasets.

Prerequisites

`{tidytof}` makes heavy use of two concepts that may be unfamiliar to R beginners. The first is the pipe (`|>`), which you can read about here. The second is "grouping" data in a `data.frame` or `tibble` using `dplyr::group_by`, which you can read about here. Most `{tidytof}` users will also benefit from a relatively in-depth understanding of the dplyr package, which has an introductory vignette here [28]:

```
vignette("dplyr")
```

Everything else should be self-explanatory for both beginner and advanced R users, though if you have *zero* background in running R code, you should read this chapter of R for Data Science by Hadley Wickham [29].

Workflow basics

Broadly speaking, `{tidytof}`'s functionality is organized to support the 3 levels of analysis inherent to single-cell data described above:

1. Reading, writing, preprocessing, and visualizing data at the level of **individual cells**
2. Identifying and describing cell **subpopulations** or **clusters**
3. Building models (for inference or prediction) at the level of **patients** or **samples**

`{tidytof}` provides functions (or "verbs") that operate at each of these levels of analysis:

- Cell-level data:
  - `tof_read_data()` reads single-cell data from FCS or CSV files on disk into a tidy data frame called a `tof_tbl`. `tof_tbl`s represent each cell as a row and each protein measurement (or other piece of information associated with a given cell) as a column.
  - `tof_preprocess()` transforms protein expression values using a user-provided function (i.e. log-transformation, centering, scaling)
  - `tof_downsample()` reduces the number of cells in a `tof_tibble` via subsampling.
  - `tof_reduce_dimensions()` performs dimensionality reduction (across columns)
  - `tof_write_data` writes single-cell data in a `tof_tibble` back to disk in the form of an FCS or CSV file.
- Cluster-level data:
  - `tof_cluster()` clusters cells using one of several algorithms commonly applied to high-dimensional cytometry data
  - `tof_metacluster()` agglomerates clusters into a smaller number of metaclusters
  - `tof_daa()` performs differential abundance analysis (DAA) for clusters or metaclusters across experimental groups
  - `tof_dea()` performs differential expression analysis (DEA) for clusters' or metaclusters' marker expression levels across experimental groups
  - `tof_extract_features()` computes summary statistics (such as mean marker expression) for each cluster. Also (optionally) pivots these summary statistics into a sample-level tidy data frame in which each row represents a sample and each column represents a cluster-level summary statistic.
  - `tof_upsample()` assigns new cells to previously-computed clusters based on the similarity between each new cell and the cells in each cluster.
- Sample-level data:
  - `tof_split_data()` splits sample-level data into a training and test set for predictive modeling
  - `tof_create_grid()` creates an elastic net hyperparameter search grid for model tuning
  - `tof_train_model()` trains a sample-level elastic net model and saves it as a `tof_model` object
  - `tof_predict()` Applies a trained `tof_model` to new data to predict sample-level outcomes
  - `tof_assess_model()` calculates performance metrics for a trained `tof_model`

{tidytof} verb syntax

With very few exceptions, `{tidytof}` functions follow a specific, shared syntax that involves 3 types of arguments that always occur in the same order. These argument types are as follows:

1. For almost all `{tidytof}` functions, the first argument is a data frame (or tibble). This enables the use of the pipe (`|>`) for multi-step calculations, which means that your first argument for most functions will be implicit (passed from the previous function using the pipe). This also means that most `{tidytof}` functions are so-called "single-table verbs," with the exception of `tof_cluster_ddpr`, which is a "two-table verb" (for details about how to use `tof_cluster_ddpr`, see the "clustering-and-metaclustering" vignette).
2. The second group of arguments are called *column specifications*, and they end in the suffix `_col` or `_cols`. Column specifications are unquoted column names that tell a `{tidytof}` verb which columns

to compute over for a particular operation. For example, the `cluster_cols` argument in `tof_cluster` allows the user to specify which column in the input data frames should be used to perform the clustering. Regardless of which verb requires them, column specifications support tidyselect helpers [30].

3. Finally, the third group of arguments for each `{tidytof}` verb are called *method specifications*, and they're comprised of every argument that isn't an input data frame or a column specification. Whereas column specifications represent which columns should be used to perform an operation, method specifications represent the details of how that operation should be performed. For example, the `tof_cluster_phenograph()` function requires the method specification `num_neighbors`, which specifies how many nearest neighbors should be used to construct the PhenoGraph algorithm's k-nearest-neighbor graph. In most cases, `{tidytof}` sets reasonable defaults for each verb's particular method specifications, but your workflows are can also be customized by experimenting with non-default values.

The following code demonstrates how `{tidytof}` verb syntax looks in practice, with column and method specifications explicitly pointed out:

```
set.seed(777L)

ddpr_data |>
  tof_preprocess() |>
  tof_cluster(
    cluster_cols = starts_with("cd"), # column specification
    method = "flowsom", # method specification,
    num_metaclusters = 5 # method specification
  ) |>
  tof_downsample(
    group_cols = .flowsom_metacluster, # column specification
    method = "constant", # method specification
    num_cells = 200 # method specification
  ) |>
  tof_plot_cells_layout(
    knn_cols = starts_with("cd"), # column specification
    color_col = .flowsom_metacluster, # column specification
    num_neighbors = 7, # method specification
    node_size = 2 # method specification
  )
```

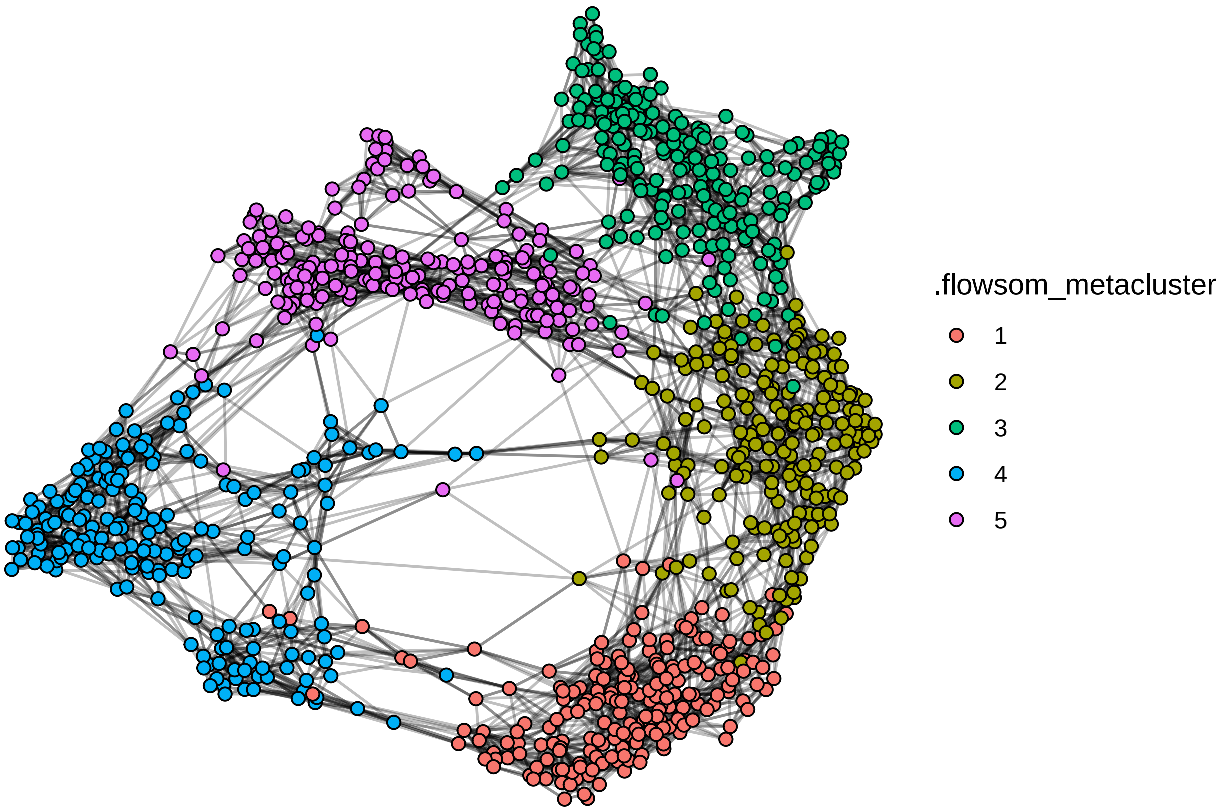

Pipelines

`{tidytof}` verbs can be used on their own or in combination with one another using the pipe (`|>`) operator. For example, here is a multistep "pipeline" that takes a built-in `{tidytof}` dataset and performs the following analytical steps:

1. Arcsinh-transform each column of protein measurements (the default behavior of the `tof_preprocess` verb
2. Cluster our cells based on the surface markers in our panel
3. Downsample the dataset such that 100 random cells are picked from each cluster
4. Perform dimensionality reduction on the downsampled dataset using tSNE
5. Visualize the clusters using a low-dimensional tSNE embedding

```
set.seed(2020L)
ddpr_data |>
  # step 1
  tof_preprocess() |>
  # step 2
  tof_cluster(
    cluster_cols = starts_with("cd"),
    method = "flowsom",
    num_metaclusters = 5L
  ) |>
  # step 3
  tof_downsample(
    group_cols = .flowsom_metacluster,
    method = "constant",
```

```
  num_cells = 500
) |>
# step 4
tof_reduce_dimensions(method = "tsne") |>
# step 5
tof_plot_cells_embedding(
  embedding_cols = contains("tsne"),
  color_col = .flowsom_metacluster
)
```

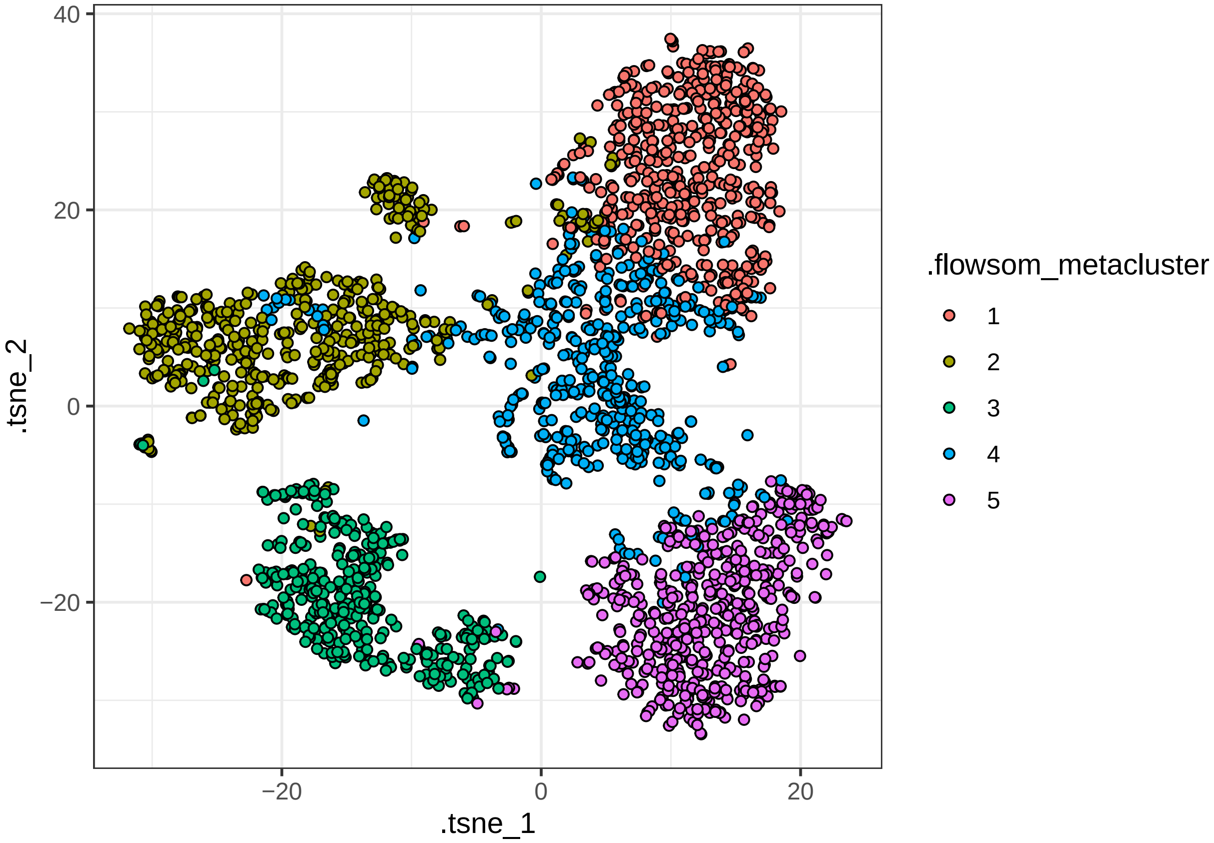

Other tips

`{tidytof}` was designed by a multidisciplinary team of wet-lab biologists, bioinformaticians, and physician-scientists who analyze high-dimensional cytometry and other kinds of single-cell data to solve a variety of problems. As a result, `{tidytof}`'s high-level API was designed with great care to mirror that of the `{tidyverse}` itself - that is, to be human-centered, consistent, composable, and inclusive for a wide userbase.

Practically speaking, this means a few things about using `{tidytof}`.

First, it means that `{tidytof}` was designed with a few quality-of-life features in mind. For example, you may notice that most `{tidytof}` functions begin with the prefix `tof_`. This is intentional, as it will allow you to use your development environment's code-completing software to search for `{tidytof}` functions easily (even if you can't remember a specific function name). For this reason, we recommend using `{tidytof}` within the RStudio development environment; however, many code editors have predictive text functionality that serves a similar function. In general, `{tidytof}` verbs are organized in such a way that your IDE's code-completion tools should also allow you to search for (and compare) related functions with relative ease. (For instance, the `tof_cluster_` prefix is used for all clustering functions, and the `tof_downsample_` prefix is used for all downsampling functions).

Second, it means that `{tidytof}` functions *should* be relatively intuitive to use due to their shared logic - in other words, if you understand how to use one `{tidytof}` function, you should understand how to use most of the others. An example of shared logic across `{tidytof}` functions is the argument `group_cols`, which shows up in multiple verbs (`tof_downsample`, `tof_cluster`, `tof_daa`, `tof_dea`, `tof_extract_features`, and `tof_write_data`). In each case, `group_cols` works the same way: it accepts an unquoted vector of column names (specified manually or using tidyselection) that should be used to group cells before an operation is performed. This idea generalizes throughout `{tidytof}`: if you see an argument in one place, it will behave identically (or at least very similarly) wherever else you encounter it.

Finally, it means that `{tidytof}` is optimized first for ease-of-use, then for performance. Because humans and computers interact with data differently, there is always a trade-off between choosing a data representation that is intuitive to a human user vs. choosing a data representation optimized for computational speed and memory efficiency. When these design choices conflict with one another, our team tends to err on the side of choosing a representation that is easy-to-understand for users even at the expense of small performance costs. Ultimately, this means that `{tidytof}` may not be the optimal tool for every high-dimensional cytometry analysis, though hopefully its general framework will provide most users with some useful functionality.

Supplementary Note 2 - Example {tidytof} workflows

In this note, we provide some example code and output demonstrating how to use {tidytof} to perform common CyTOF data analysis tasks. For additional, exhaustive details for each of {tidytof}'s functions, see the package website.

Accessing the data for this vignette

First, we will download a dataset originally collected for the development of the CITRUS algorithm. These data are available in the `{HDCytoData}` package, which is available on Bioconductor and can be accessed with the following command:

```
if (!requireNamespace("BiocManager", quietly = TRUE)) {
  install.packages("BiocManager")
}

BiocManager::install("HDCytoData")
```

To load the CITRUS data into our current R session, we can call a function from the `{HDCytoData}`, which will provide it to us in a format from the `{flowCore}` package (called a "flowSet"). To convert this into a tidy tibble, we can use `{tidytof}` built-in method for converting {flowCore} objects into `tof_tbl`'s .

```
citrus_raw <- HDCytoData::Bodenmiller_BCR_XL_flowSet()

citrus_data <-
  citrus_raw |>
  as_tof_tbl(sep = "_")
```

Thus, we can see that `citrus_data` is a `tof_tbl` with 172791 cells (one in each row) and 39 pieces of information about each cell (one in each column).

We can also extract some metadata from the raw data and join it with our single-cell data using some functions from the `tidyverse`:

```
citrus_metadata <-
  tibble(
    file_name = as.character(flowCore::pData(citrus_raw)[[1]]),
    sample_id = 1:length(file_name),
    patient = stringr::str_extract(file_name, "patient[:digit:]"),
    stimulation = stringr::str_extract(file_name, "(BCR-XL)|Reference")
  ) |>
  mutate(
    stimulation = if_else(stimulation == "Reference", "Basal", stimulation)
  )

citrus_metadata |>
  head()
```

Thus, we now have sample-level information about which patient each sample was collected from and which stimulation condition ("Basal" or "BCR-XL") each sample was exposed to before data acquisition.

Finally, we can join this metadata with our single-cell `tof_tbl` to obtain the cleaned dataset.

```
citrus_data <-
  citrus_data |>
  left_join(citrus_metadata, by = "sample_id")
```

After these data cleaning steps, we now have `citrus_data`, a `tof_tbl` containing cells collected from 8 patients. Specifically, 2 samples were taken from each patient: one in which the cells' B-cell receptors

were stimulated ("BCR-XL") and one in which they were not ("Basal"). In `citrus_data`, each cell's patient of origin is stored in the `patient` column, and each cell's stimulation condition is stored in the `stimulation` column. In addition, the `population_id` column stores information about cluster labels that were applied to each cell using a combination of FlowSOM clustering and manual merging (for details, run `?HDCytoData::Bodenmiller_BCR_XL` in the R console).

Analyzing single-cell data

As a `tof_tbl`, `citrus_data` has access to all {dplyr} functions for general data cleaning. For example, we might want to remove some columns that are either redundant or represent measurements that we're not interested in using the `select()` function. In addition, we might want to convert some categorical columns initially represented as numeric vectors into character vectors so that we don't accidentally use them for computation using the `mutate()` function. We can also rename each of our protein measurement columns to omit the metal names using the `rename()` function in order to make column selection a bit easier.

```
citrus_data <-
  citrus_data |>
  select(
    -Time,
    -Cell_length,
    -contains("DNA"),
    -contains("Dd"),
    -contains("BC", ignore.case = FALSE),
    -patient_id,
    -group_id
  ) |>
  mutate(
    population_id = as.character(population_id),
    sample_id = as.character(sample_id)
  ) |>
  rename_with(
    .fn = ~ str_remove(.x, pattern = "_.+$"),
    .cols = c(-file_name, -population_id, -sample_id)
  )
```

We can also apply a common variance-stabilizing transformation (the arcsinh transformation) to each protein measurement column of `citrus_data` using {tidytof}'s `tof_preprocess()` verb:

```
citrus_data <-
  citrus_data |>
  tof_preprocess()
```

From here, we can try out several workflows for analyzing and visualizing `citrus_data` using various {tidytof} verbs:

```
citrus_data |>
  # cluster cells based on their surface phenotype using flowSOM
  tof_cluster(cluster_cols = starts_with("CD"), method = "flowsom") |>
  # downsample cells from each flowsom cluster
  tof_downsample(group_cols = population_id, num_cells = 100, method = "constant") |>
  # make a force-directed layout plot
  tof_plot_cells_layout(knn_cols = starts_with("CD"), color_col = .flowsom_metacluster)
```

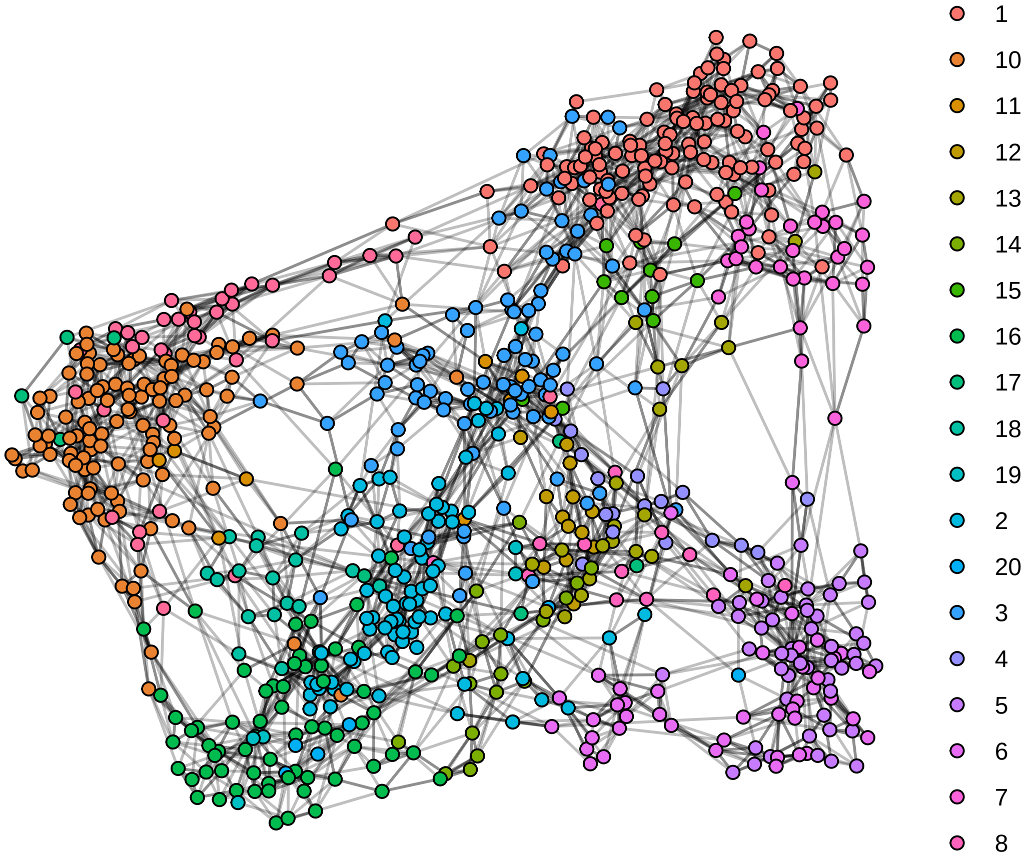


```
citrus_data |>
  # downsample within each (pre-identified) cell subpopulation
  tof_downsample(group_cols = population_id, num_cells = 500, method = "constant") |>
  # perform dimensionality reduction using UMAP
  tof_reduce_dimensions(method = "umap") |>
  # make a plot
  tof_plot_cells_embedding(
    embedding_cols = contains("UMAP"),
    color_col = population_id
  )
```

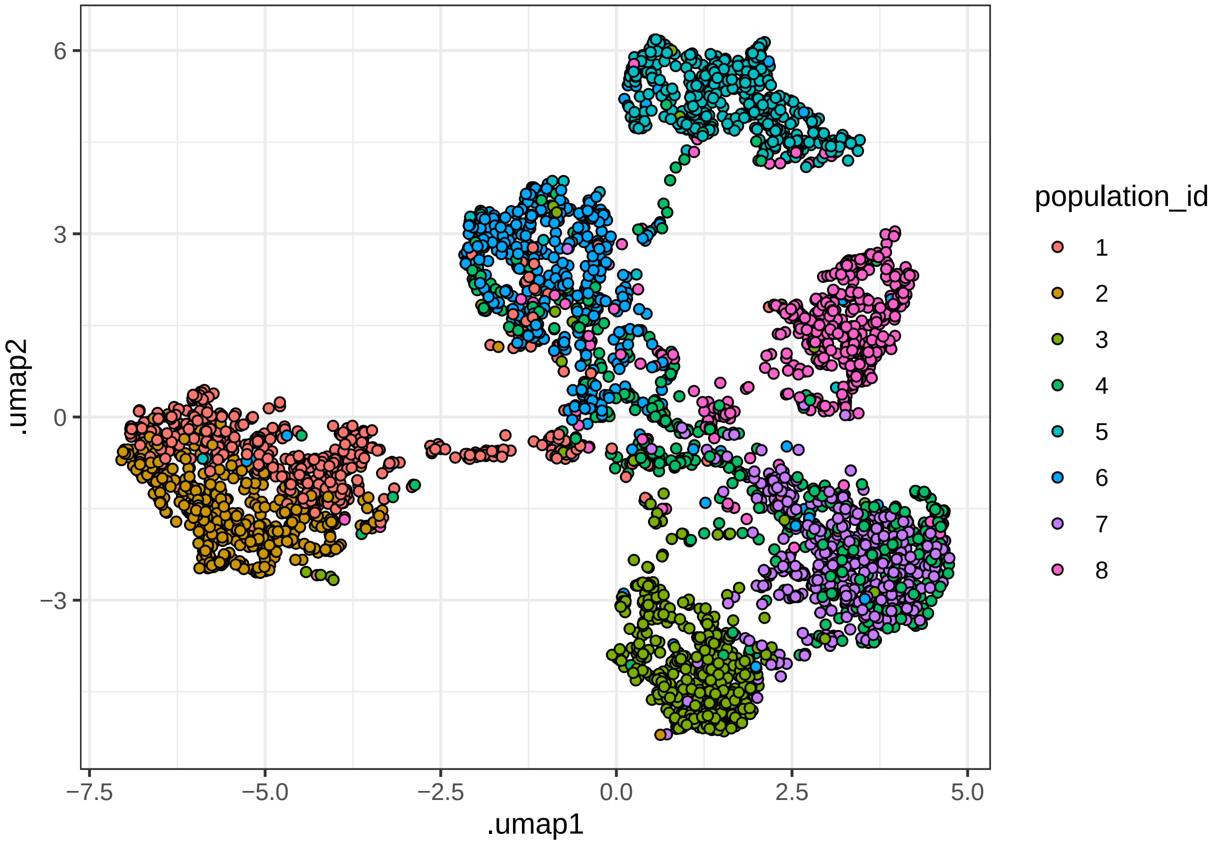


```
citrus_data |>
  # downsample within each (pre-identified) cell subpopulation
  tof_downsample(group_cols = population_id, num_cells = 500, method = "constant") |>
  # perform dimensionality reduction using tsne
  tof_reduce_dimensions(method = "tsne") |>
  # make a plot
  tof_plot_cells_embedding(
    embedding_cols = contains("tsne"),
    color_col = population_id
  )
```

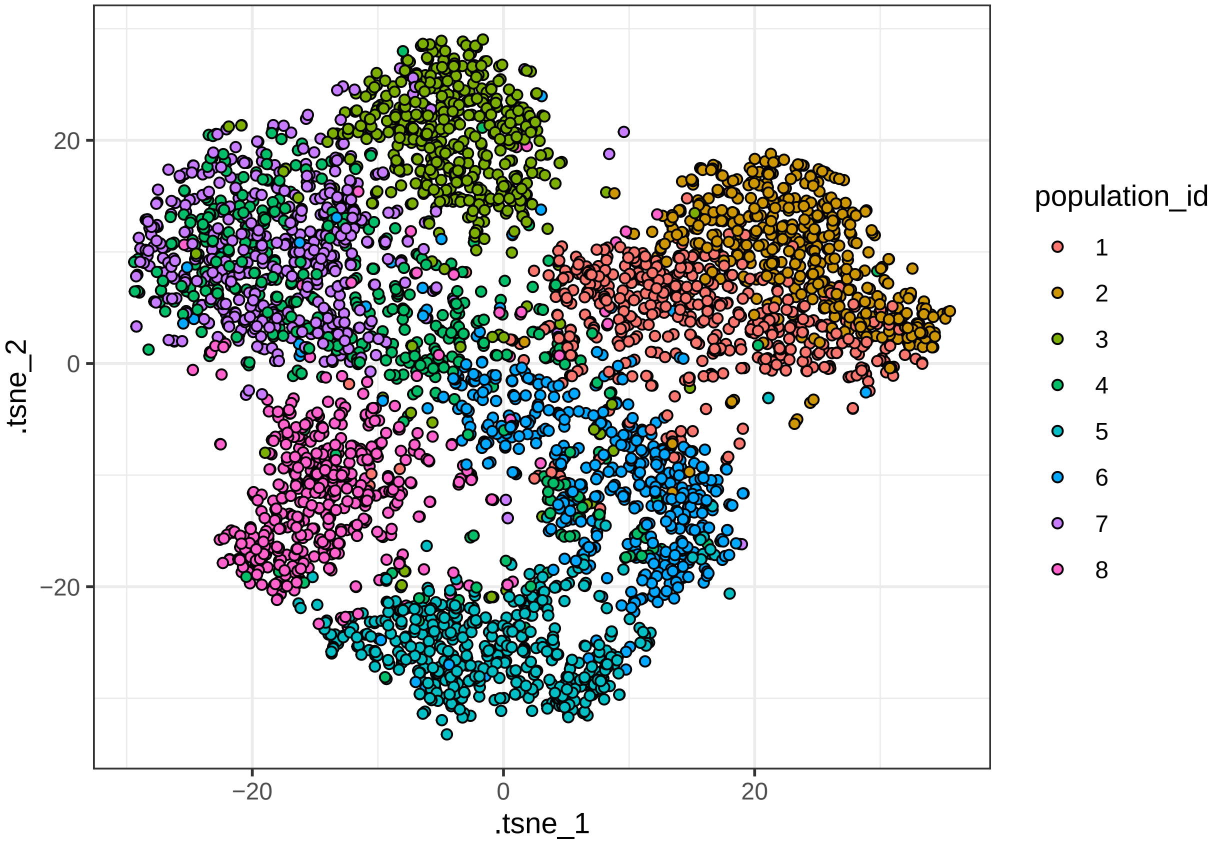

Analyzing cluster-level data

```
citrus_data |>
  # perform differential abundance analysis using paired t-tests with
  # multiple hypothesis correction
  tof_analyze_abundance_ttest(
    cluster_col = population_id,
    group_cols = c(patient, stimulation),
    effect_col = stimulation,
    test_type = "paired"
  )
```

```
citrus_data |>
  # perform differential expression analysis using paired t-tests with
  # multiple hypothesis correction
  tof_analyze_expression_ttest(
    cluster_col = population_id,
    marker_cols = where(is.double),
    group_cols = c(patient, stimulation),
    effect_col = stimulation,
    test_type = "paired"
  ) |>
  # select only the significant cluster-marker pairs
  dplyr::filter(significant == "*")
```

```
citrus_data |>
  # compute the mean of each marker for each cluster in each patient
```

```r
  tof_extract_central_tendency(
    cluster_col = population_id,
    group_cols = patient,
    marker_cols = where(is.double),
    central_tendency_function = mean
  )
```

```r
citrus_data |>
  # compute the abundance of each cluster in each  patient
  tof_extract_proportion(
    cluster_col = population_id,
    group_cols = patient
  )
```

```r
citrus_data |>
  # compute the median of each marker for each cluster in each patient
  tof_extract_central_tendency(
    cluster_col = population_id,
    group_cols = patient,
    marker_cols = where(is.double),
    central_tendency_function = median,
    # request in long format, i.e. suitable for {ggplot2}
    format = "long"
  ) |>
  # only look at pSTAT1
  dplyr::filter(stringr::str_detect(channel, "pStat1")) |>
  # make a plot using {ggplot2}
  ggplot(aes(x = population_id, y = values, fill = patient)) +
  geom_point(shape = 21) +
  labs(subtitle = "median pSTAT1 expression in each patient") +
  theme_bw()
```

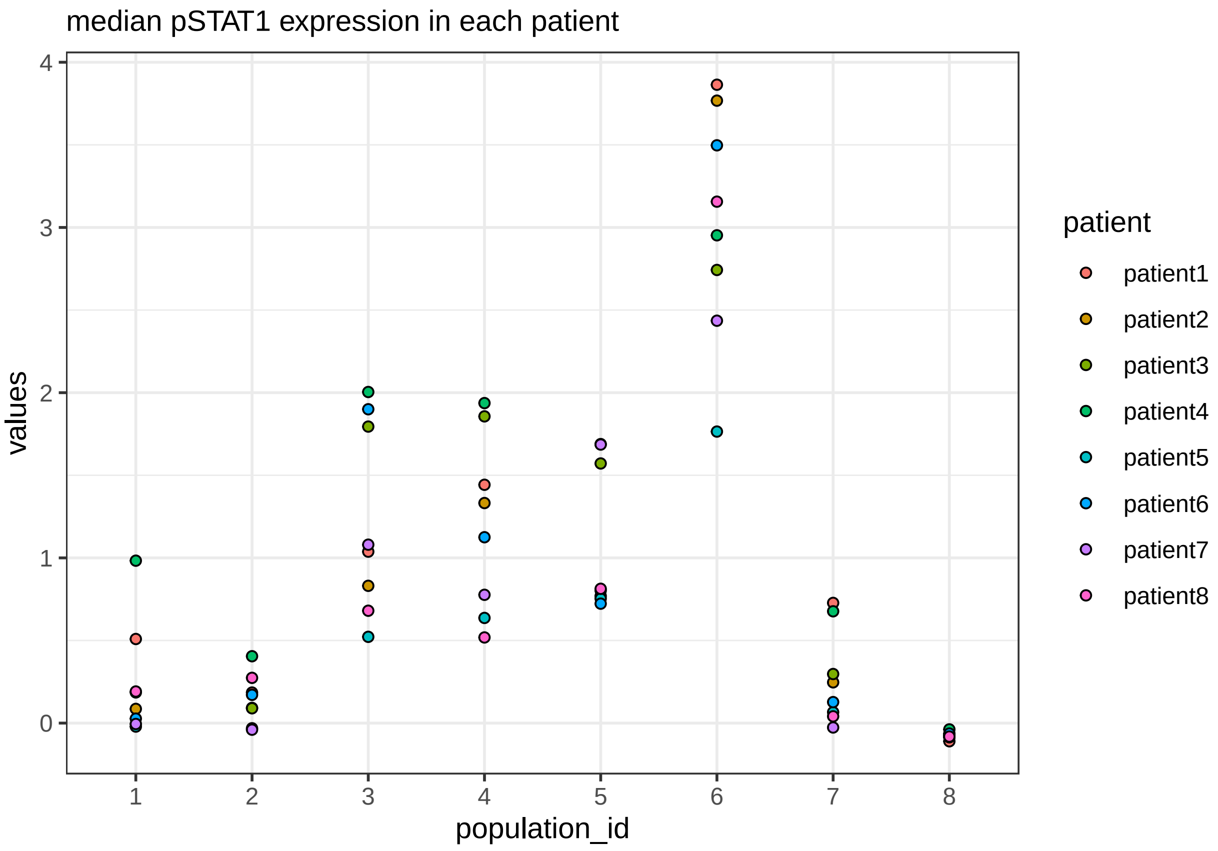

Analyzing sample-level data

Using `citrus_data`, we can build a predictive model of for which samples are in the stimulation condition and which ones are in the basal condition solely using signaling phenotypes.

```
signaling <-
  c("pNFkB", "pp38", "pStat5", "pAkt", "pStat1", "pStat3", "pBtk", "pS6")

model_result <-
  citrus_data |>
  # compute sample-level features from the input single-cell data using only
  # signaling columns (ones most relevant to the stimulation condition)
  tof_extract_features(
    cluster_col = population_id,
    group_cols = c(patient, stimulation),
    lineage_cols = all_of(signaling),
    signaling_cols = all_of(signaling)
  ) |>
  # encode outcome variable as a factor
  mutate(stimulation = as.factor(stimulation)) |>
  # split data into 3 cross-validation folds
  tof_split_data(num_cv_folds = 3) |>
  # train logistic regression model with lasso regularization
  # using 3-fold cross validation
  tof_train_model(
    predictor_cols = c(-patient, -stimulation),
    response_col = stimulation,
```

```r
    model_type = "two-class",
    remove_zv_predictors = TRUE,
    impute_missing_predictors = TRUE,
    hyperparameter_grid = tof_create_grid(
      mixture_values = 1,
      penalty_values = c(0.001, 0.01, 0.1, 0.4)
    )
  )

# assess the model's performance on the full dataset
model_metrics <-
  model_result |>
  tof_assess_model()


model_result |>
  tof_plot_model()
```

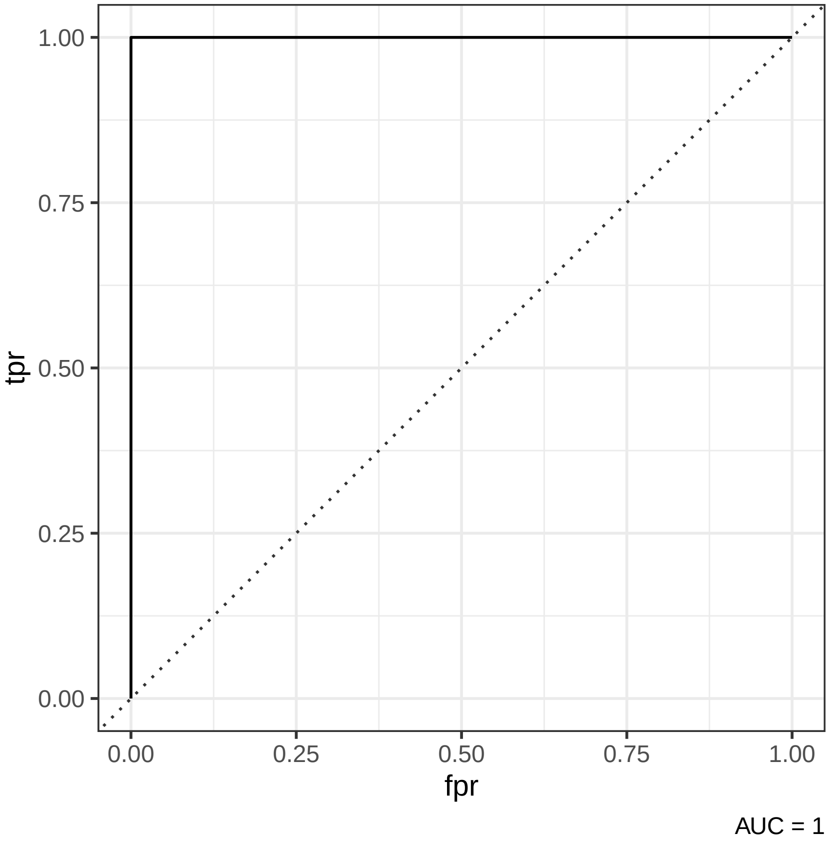

References

1. Qiu P, Simonds EF, Bendall SC, Gibbs KD, Bruggner RV, Linderman MD, et al. Extracting a cellular hierarchy from high-dimensional cytometry data with SPADE. Nat Biotechnol. 2011;29:886–91.
2. Amir el-AD, Davis KL, Tadmor MD, Simonds EF, Levine JH, Bendall SC, et al. viSNE enables visualization of high dimensional single-cell data and reveals phenotypic heterogeneity of leukemia. Nat Biotechnol. 2013;31:545–52.
3. McInnes L, Healy J, Saul N, Großberger L. UMAP: Uniform manifold approximation and projection. Journal of Open Source Software. 2018;3:861.
4. Good Z, Sarno J, Jager A, Samusik N, Aghaeepour N, Simonds EF, et al. Single-cell developmental classification of B cell precursor acute lymphoblastic leukemia at diagnosis reveals predictors of relapse. Nat Med. 2018;24:474–83.
5. Levine JH, Simonds EF, Bendall SC, Davis KL, Amir el-AD, Tadmor MD, et al. Data-Driven Phenotypic Dissection of AML Reveals Progenitor-like Cells that Correlate with Prognosis. Cell. 2015;162:184–97.
6. Quintelier K, Couckuyt A, Emmaneel A, Aerts J, Saeys Y, Van Gassen S. Analyzing high-dimensional cytometry data using FlowSOM. Nat Protoc. 2021;16:3775–801.
7. Samusik N, Good Z, Spitzer MH, Davis KL, Nolan GP. Automated mapping of phenotype space with single-cell data. Nat Methods. 2016;13:493–6.
8. Wilkerson, D. M, Hayes, Neil D. ConsensusClusterPlus: A class discovery tool with confidence assessments and item tracking. Bioinformatics. 2010;26:1572–3.
9. Weber LM, Nowicka M, Soneson C, Robinson MD. diffcyt: Differential discovery in high-dimensional cytometry via high-resolution clustering. Commun Biol. 2019;2:183.
10. Nowicka M, Krieg C, Crowell HL, Weber LM, Hartmann FJ, Guglietta S, et al. CyTOF workflow: differential discovery in high-throughput high-dimensional cytometry datasets. F1000Res. 2017;6:748.
11. Bruggner RV, Bodenmiller B, Dill DL, Tibshirani RJ, Nolan GP. Automated identification of stratifying signatures in cellular subpopulations. Proc Natl Acad Sci U S A. 2014;111:E2770–2777.
12. Silge J, Chow F, Kuhn M, Wickham H. Rsample: General resampling infrastructure. 2021.
13. Friedman J, Hastie T, Tibshirani R. Regularization paths for generalized linear models via coordinate descent. Journal of Statistical Software. 2010;33:1–22.
14. Simon N, Friedman J, Hastie T, Tibshirani R. Regularization paths for cox's proportional hazards model via coordinate descent. Journal of Statistical Software. 2011;39:1–13.
15. R Core Team. R: A language and environment for statistical computing. Vienna, Austria: R Foundation for Statistical Computing; 2021.
16. Ellis B, Haaland P, Hahne F, Le Meur N, Gopalakrishnan N, Spidlen J, et al. flowCore: flowCore: Basic structures for flow cytometry data. 2021.
17. Chen H, Lau MC, Wong MT, Newell EW, Poidinger M, Chen J. Cytofkit: A Bioconductor Package for an Integrated Mass Cytometry Data Analysis Pipeline. PLoS Comput Biol. 2016;12:e1005112.
18. Opzoomer JW, Timms JA, Blighe K, Mourikis TP, Chapuis N, Bekoe R, et al. ImmunoCluster provides a computational framework for the nonspecialist to profile high-dimensional cytometry data. Elife. 2021;10.
19. Ashhurst TM, Marsh-Wakefield F, Putri GH, Spiteri AG, Shinko D, Read MN, et al. Integration, exploration, and analysis of high-dimensional single-cell cytometry data using Spectre. Cytometry A. 2021.
20. Amezquita R, Lun A, Becht E, Carey V, Carpp L, Geistlinger L, et al. Orchestrating single-cell analysis with bioconductor. Nature Methods. 2020;17:137–45.
21. Dowle M, Srinivasan A. Data.table: Extension of 'data.frame'. 2021.
22. Mersmann O. Microbenchmark: Accurate timing functions. 2021.
23. Wickham H. Lobstr: Visualize r data structures with trees. 2019.
24. Müller K, Walthert L. Styler: Non-invasive pretty printing of r code. 2021.
25. Kuhn M. Embed: Extra recipes for encoding predictors. 2021.
26. Kuhn M, Wickham H. Recipes: Preprocessing and feature engineering steps for modeling. 2021.
27. Bendall SC, Simonds EF, Qiu P, Amir el-AD, Krutzik PO, Finck R, et al. Single-cell mass cytometry of differential immune and drug responses across a human hematopoietic continuum. Science. 2011;332:687–96.
28. Wickham H, François R, Henry L, Müller K. Dplyr: A grammar of data manipulation. 2022.
29. Wickham H, Grolemund G. R for data science: Import, tidy, transform, visualize, and model data. 1st edition. O'Reilly Media, Inc.; 2017.
30. Henry L, Wickham H. Tidyselect: Select from a set of strings. 2021.
